# A possible beneficial effect of *Bacteroides* on faecal lipopolysaccharide activity and cardiovascular diseases

**DOI:** 10.1038/s41598-020-69983-z

**Published:** 2020-08-03

**Authors:** Naofumi Yoshida, Tomoya Yamashita, Shigenobu Kishino, Hikaru Watanabe, Kengo Sasaki, Daisuke Sasaki, Tokiko Tabata, Yuta Sugiyama, Nahoko Kitamura, Yoshihiro Saito, Takuo Emoto, Tomohiro Hayashi, Tomoya Takahashi, Masakazu Shinohara, Ro Osawa, Akihiko Kondo, Takuji Yamada, Jun Ogawa, Ken-ichi Hirata

**Affiliations:** 1 Division of Cardiovascular Medicine, Department of Internal Medicine, Kobe University Graduate School of Medicine, 7-5-1 Kusunoki-cho, Chuo-ku, Kobe, 6500017 Japan; 2 Division of Applied Life Science, Graduate School of Agriculture, Kyoto University, Kyoto, 6068502 Japan; 3 School and Graduate School of Bioscience and Biotechnology, Tokyo Institute of Technology, Tokyo, 1528550 Japan; 4 Graduate School of Science, Technology and Innovation, Kobe University, Kobe, 6578501 Japan; 5 Division of Epidemiology, Department of Community Medicine and Social Healthcare Science, Kobe University Graduate School of Medicine, Kobe, 6500017 Japan; 6 Department of Bioresource Science, Graduate School of Agricultural Science, Kobe University, Kobe, 6578501 Japan

**Keywords:** Immunology, Microbiology, Cardiology

## Abstract

Faecal lipopolysaccharides (LPS) have attracted attention as potent elements to explain a correlation between the gut microbiota and cardiovascular disease (CVD) progression. However, the underlying mechanism of how specific gut bacteria contribute to faecal LPS levels remains unclear. We retrospectively analysed the data of 92 patients and found that the abundance of the genus *Bacteroides* was significantly and negatively correlated with faecal LPS levels. The controls showed a higher abundance of *Bacteroides* than that in the patients with CVD. The endotoxin units of the *Bacteroides* LPS, as determined by the limulus amoebocyte lysate (LAL) tests, were drastically lower than those of the *Escherichia coli* LPS; similarly, the *Bacteroides* LPS induced relatively low levels of pro-inflammatory cytokine production and did not induce sepsis in mice. Fermenting patient faecal samples in a single-batch fermentation system with *Bacteroides* probiotics led to a significant increase in the *Bacteroides* abundance, suggesting that the human gut microbiota could be manipulated toward decreasing the faecal LPS levels. In the clinical perspective, *Bacteroides* decrease faecal LPS levels because of their reduced LAL activity; therefore, increasing *Bacteroides* abundance might serve as a novel therapeutic approach to prevent CVD via reducing faecal LPS levels and suppressing immune responses.

## Introduction

Increasing evidence suggests that a strong correlation exists between the gut microbiota and development of many diseases, such as coronary artery disease (CAD), heart failure (HF), obesity and related metabolic diseases, and rheumatoid arthritis^1–6^. Next-generation sequencing techniques and multi-omics approaches have revealed the function of the gut microbiota and improved understanding of the mechanisms underlying disease progression^7–10^. For instance, the short-chain fatty acid metabolites produced by the gut microbiota are known to play a central immune-metabolic role^11^, where trimethylamine and trimethylamine *N*-oxide are associated with cardiovascular disease^12^. In addition, the gut microbial lipopolysaccharide (LPS) has recently attracted attention, because chronic immune cell activation is, in part, caused by the LPS-mediated stimulation of toll-like receptor 4 (TLR4) in the gu^13,14^. Until now, researchers have focused on plasma LPS, and have reported that plasma LPS plays an important role in the process of activating pro-inflammatory cytokines during heart failure, and may be used to predict the development of atherosclerosis and other major advanced cardiac events^15–17^. In this context, faecal LPS levels are now considered potent elements that may be used to explain a strong correlation between the gut microbiota and disease progression and as a novel dysbiosis marker or risk factor for inflammatory diseases^18–22^. However, how specific gut bacteria contribute to faecal LPS levels remains unclear.

Interestingly, each bacterial species has distinct LPS structures^13,14,23–25^. In particular, structural variations in the lipid A moiety, characterised by diversity in the acylation pattern, are associated with different immunogenicity. Specifically, the penta- and tetra-acylated lipid A moieties of the *Bacteroides* species elicits reduced TLR4 responses compared to the hexa-acylated lipid A moiety of *Escherichia coli*^13,14^. A recent study indicates that a variety of innate immune responses induced by structural differences in lipid A moiety are associated with the incidence of autoimmune diseases^13^. Although the limulus amoebocyte lysate (LAL) tests are the gold standard for measuring LPS levels, it is important to remember that LPS demonstrates its own unique LAL activity due to the structural differences in lipid A moiety,therefore, faecal LPS levels measured by the LAL tests do not correctly reflect the absolute mass of LPS^26^. In other words, the variation of LAL activity among bacterial lipid A types might play a key role in the interpretation of faecal LPS levels. However, the impact of structural differences between the lipid A moieties of different gut bacteria on faecal LPS levels has not yet been measured using the LAL tests.

Here, we aimed to elucidate which gut bacteria contribute the most to faecal LPS levels measured by LAL tests and the reason for this impact. We also aimed to investigate whether probiotics manipulate the human gut microbiota toward decreasing faecal LPS levels using a single-batch anaerobic culturing system, Kobe University Human Intestinal Microbiota Model (KUHIMM). The KUHIMM is capable of hosting more than 500 bacterial species and can effectively and accurately mimic the healthy human gut microbiota and a patients’ impaired colonic microbiota; this system simulates the human gut microbiota metagenomically, and can overcome ethical and safety considerations of human intervention clinical trials^27–29^. We fermented patient faecal samples in the KUHIMM with probiotics to facilitate evaluation of the effect of probiotics on the gut microbiota before clinical studies.

## Methods

### Retrospective analysis: study design and participants

**Figure 1:**
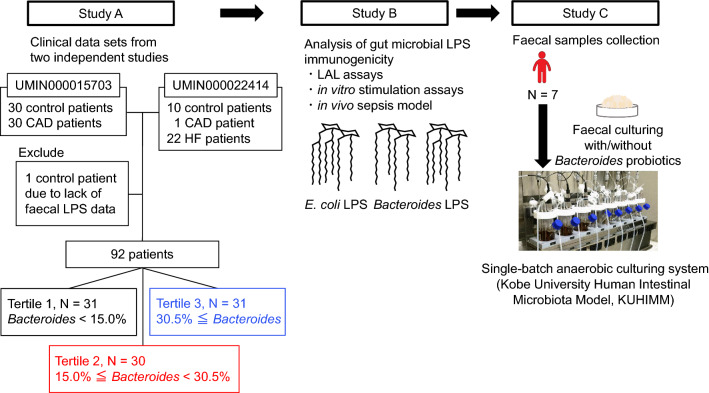
Overview of experimental design. This investigation consisted of three separate studies: study A, study B, and study C. The goal of study A was to clarify which and how specific gut bacteria contribute to faecal LPS levels using the human data. The goal of study B was to examine the LPS bioactivity of the gut bacteria determined in study A in vitro and in vivo. The goal of study C was to elucidate that probiotics could change the gut flora of patients to symbiotic states toward decreasing their faecal LPS levels prior to the clinical trials. *CAD* coronary artery disease; *E. coli*, *Escherichia coli*; *HF* heart failure; *LAL* Limulus amoebocyte lysate; *LPS* lipopolysaccharide.

In our retrospective analysis, we accessed 93 clinical data sets from two independent studies (UMIN000015703 and UMIN000022414) (Fig. 1)^1,30^. As one patient was excluded because of the lack of detectable faecal LPS levels, we finally analysed the data of 92 patients. All study participants were recruited at Kobe University Hospital between October 2014 and April 2017. Faecal samples were collected while participants were hospitalised and consumed a hospital diet for more than 1 day. Patients with HF were admitted for de novo acute decompensated HF or acute worsening of chronic HF. HF was defined based on the modified Framingham criteria^31^. The CAD patients, who underwent coronary artery bypass graft (CABG) surgery or percutaneous coronary intervention, demonstrated > 75% stenosis on diagnostic coronary angiography, with preserved left ventricular ejection fraction and no history of HF. Patients with acute coronary syndrome were excluded. Control patients, who were hospitalised at the same hospital, had hypertension, diabetes mellitus, or dyslipidemia as per the guidelines^32^, but no history of CAD and HF. Patients who had undergone antibiotic treatment within a month before admission and during hospitalization were excluded. All participants provided written informed consent, and the study was conducted according to the principles of the Declaration of Helsinki. This study was approved by the Medical Ethics Committee at the Kobe University (No. B190088) and was registered with the University Hospital Medical Information Network (UMIN) Clinical Trials Registry (UMIN000036752).

### Faecal LPS levels

Faecal supernatant was obtained according to a previously described protocol, with some modifications^18^. Briefly, the faecal samples were suspended in sterile PBS to a concentration of 1 g per 10 mL and vortexed mildly to avoid disruption of bacterial cells. After centrifugation for 15 min at 3,000 rpm, the supernatant was collected and sterilised by filtration through a 0.45-μm filter, followed by re-filtration through a 0.22-μm filter and inactivation for 15 min at 90 °C. The faecal LPS levels were determined using the LAL assay (#K50-643 J; Lonza Inc., Basel, Switzerland), according to the manufacturer’s instructions. The LPS measurements were performed in pyrogen-free glass tubes, Eppendorf tubes, and 96-well plates.

### DNA extraction, 16S rRNA gene amplification, and pyrosequencing

The collected faecal samples were stored at − 80 °C. DNA extraction from human faecal samples was performed by the Nihon Gene Research Laboratories Inc., according to a previously established procedure^33^.

Parts of the 16S rRNA genes (the V3–V4 region, corresponding to positions 342 to 806 in the *E. coli* numbering system) were PCR-amplified using our non-degenerate universal primer set of 342F and 806R. A detailed description of the primer set and PCR conditions is available elsewhere^34^. After adding the sequencing adapters, the amplicons were sequenced by Takara Bio Inc. using an Illumina MiSeq platform (Illumina Inc., San Diego, CA) according to the manufacturer’s protocol. To make the bacterial composition matrix, we used USEARCH version 10.0.240. Our previous protocol was also used to select high-quality 16S rRNA gene amplicon sequences generated using Trimmomatic^35^ version 0.33 with the parameters ‘LEADING:17 TRAILING:17 AVGQUAL:25 MINLEN:100’. The remaining reads were processed using the -fastq_mergepairs command of USEARCH, with default parameters. Next, we removed the sequences lacking the primer region using Tagcleaner version 0.16, with parameters “-tag5 CTACGGGGGGCAGCAG -mm5 3 -tag3 AGATACCCCGGTAGTCC -mm3 3 -nomatch 3”. After removing the primer, the sequences with unknown nucleotides (N) were removed using an in-house python script. To remove the PhiX reads, we used the -filter_phix command of USEARCH. Thereafter, we removed the short sequences (< 300 nucleotides) using the USEARCH command—sort_by_length, with the parameter ‘-minseqlength 300’. Finally, we generated operational taxonomic unit (OTU) tables using the UPARSE algorithm (-fastx_unique and otu_cluster commands with the parameter ‘-minsize 1’). The representative sequences of each OTU were annotated to bacterial genus using Ribosomal Database Project (RDP) Classifier version 2.12, with a bootstrap value ≥ 0.5. Moreover, we annotated each representative sequence of each OTU to the reference database Silva Living Tree Project version 123^36^ using BLASTN version 2.2.25, with the identity threshold ≥ 97% and coverage ≥ 80%.

### Animals

Wild-type C57BL/6J mice were purchased from CLEA Japan (Tokyo, Japan), and *Tlr4*^−/−^ mice on a C57BL/6J background were purchased from Charles River Japan (Yokohama, Japan). The mice were housed in a specific pathogen-free animal facility at the Kobe University Institute. They were fed a standard chow diet (CE-2, CLEA, Tokyo, Japan) and water ad libitum under a strict 12-h light cycle. The *E. coli* LPS, purified by phenol extraction, was purchased from Sigma-Aldrich (St. Louis, MO, #L2630), and *Bacteroides* cells were prepared for LPS purification as outlined in Sect. "LPS extraction from *Bacteroides vulgatus* and *B. dorei*". The 8-week-old wild-type and *Tlr4*^−/−^ mice were treated with LPS intraperitoneally to induce septic shock^37^. Survival was monitored daily after LPS was injected. This study was approved by the Animal Ethics Committee at the Kobe University (P190706), and was performed according to the Guidelines for Animal Experiments in effect at Kobe University School of Medicine.

### LPS extraction from *Bacteroides vulgatus* and *B. dorei*

LPS was purified by hot phenol-water method as previously described with minor modification^38^. Bacteria were cultivated in Gifu anaerobic medium (Nissui pharmaceutical, Tokyo, Japan) at 37 °C for 24 h under anaerobic conditions. Subsequently, 100 mL of cultured bacteria were washed twice with 15 mL of PBS containing 0.15 mM CaCl_2_ and 0.5 mM MgCl_2_. The washed cells were resuspended in 8 mL of PBS and disrupted by sonication on ice for 10 min (Insonator model 201 M; KUBOTA, Tokyo). Thereafter, 80 μL of proteinase K (10 mg/mL; Wako Pure Chemical, Osaka, Japan) was added to the cell suspension and incubated for 1 h at 65 °C. This was followed by treatment with DNase I (20 μg/mL; FUJIFILM Wako Pure Chemical, Osaka, Japan) and RNase A (40 μg/mL; Macherey–nagel, Düren, Germany) in the presence of 0.81 mM MgSO_4_ and 4 μL/mL chloroform at 37 °C overnight with shaking. Equal volume of phenol was added to the cell suspension and shook for 15 min at 65 °C. The resulting mixture was centrifuged (8,500×*g*, 4 °C, 15 min), and the water layer was collected. Sodium acetate was added to the water layer to a final concentration of 0.5 M. Ethanol was then added 10 times the volume of the water layer and placed at − 20 °C overnight. The resulting mixture was centrifuged (2000×*g*, 4 °C, 10 min), and the pellet was resuspended in water. The residual phenol was removed by dialysis against water at 4 °C using Spectra/Por 6 (MWCO 1,000; Spectrum Laboratories Inc., Ranch Dominquez, CA, USA). After purification, the materials were separated by SDS-PAGE using 4% stacking gel and 15% separating gel and stained with silver stain (Silver Stain Kit II, Wako Pure Chemical) and Coomassie brilliant blue to confirm purity. The signals were detected using ApeosPort-V C4475 (Fujixerox, Tokyo, Japan) with molecular size marker (EzStandard AE-1440, ATTO, Tokyo, Japan). No image processing software was used. The purified LPS was lyophilised and stored at − 20 °C.

### In vitro cytokine stimulation assays

RAW 264.7 macrophages were cultured in RPMI-1640 medium supplemented with 10% foetal bovine serum. The cells (1 × 10^4^) were plated in 96-well flat-bottom plates (Corning Costar, Corning, NY) and incubated in the presence of *E. coli* LPS or *Bacteroides* LPS for 12, 24, 48, and 72 h at 37 °C with 5% CO_2_. The cytokine levels were analysed using the Cytometric Bead Array Kit (BD Biosciences, San Diego, CA, USA), according to the manufacturer’s instructions.

### Faecal culture in the KUHIMM

Faecal samples were collected from seven patients with CAD during their hospital stay at Kobe University Hospital between August 2018 and May 2019. Each faecal sample was collected with an anaerobic culture swab (#212550; BD BBL, NJ, USA) and fermented for 24 h^27^. Patients who had undergone antibiotic treatment within a month before admission and during hospitalization were excluded. The sample size was calculated using the R software (power = 0.9, significance level = 0.05, mean difference = 8, SD = 5; N = 7 per group). All study participants provided written informed consent, and the study was conducted according to the principles of the Declaration of Helsinki. This study was approved by the Medical Ethics Committee at the Kobe University (No.160191) and was registered with the UMIN Clinical Trials Registry (UMIN000024555).

The Kobe University Human Intestinal Microbiota Model (KUHIMM) is a small-scale multi-channel fermenter (Bio Jr.8; ABLE, Tokyo, Japan) composed of eight parallel and independent anaerobic culturing vessels (Fig. 1)^27–29^. Each vessel contained 100 mL Gifu anaerobic medium (Nissui Pharmaceutical Co, Tokyo, Japan) at pH 6.5. The cultures were kept at 37 °C with consistent stirring at 300 rpm. Filtered N_2_:CO_2_ (80:20) gas was supplied continuously at a constant flow rate of 15 mL/min to maintain anaerobiosis. The faecal samples were suspended in 2 mL of 0.1 M phosphate buffer (2:1 of NaH_2_PO_4_ and 0.1 M Na_2_HPO_4_) supplemented with 1% L-ascorbic acid. One hundred microliters of faecal suspension was inoculated in one vessel (culture group). Immediately after faecal inoculation, 1 × 10^8^ cells each of *B. vulgatus* and *B. dorei* were added to the vessel (Bacteroides group). The faecal cultures were fermented for 48 h.

### Gut microbial analysis of faecal culture

The gut microbial profiles of faecal culture samples were determined by 16S rRNA gene sequencing, as described in detail elsewhere^28,29^. Briefly, the V3-V4 region of the 16S rRNA gene was amplified, and index primers were added to the gene-specific sequences. After PCR, the products were purified and eluted in 25 μL of 10 mM Tris buffer. The 16S rRNA genes and an internal control (PhiX control v3; Illumina) were subjected to 600 cycles of paired-end sequencing using a MiSeq sequencer and reagent kit v3 (Illumina, Inc.). The PhiX sequences were removed, and the paired-end reads with Q scores ≥ 20 were joined using the MacQIIME software, version 1.9.1 (Werner Lab, Cortland, NY, USA). The UCLUST algorithm was used to cluster the filtered sequences into OTUs based on a similarity threshold of ≥ 97%. Chimeric sequences were identified and removed from the library using ChimeraSlayer. Representative sequences from each OTU were taxonomically classified using the GreenGenes taxonomic database and the RDP Classifier.

### Statistical analysis

Statistical analyses were performed using Prism version 7.0 (GraphPad Software; San Diego, CA), R version 3.1.0, and JMP version 14 (SAS Institute, Cary, NC). The Shapiro–Wilk test was used to determine the normality of data. The results were expressed as mean ± standard error of the mean for the normally distributed data and as median ± interquartile range (25th–75th percentiles) for the non-normally distributed data. The significance of differences between the two groups was evaluated using the two-tailed Student’s *t*-test for the normally distributed data and Mann–Whitney U-test for the non-normally distributed data. Differences in continuous parameters among the three groups were calculated using one-way analysis of variance for the parametric data and Kruskal–Wallis test for the non-normally distributed data. The matched-pair samples were compared using the Wilcoxon signed-rank test. The Fisher’s exact test or Chi-square test was used to compare categorical variables. The Kaplan–Meier survival curves were constructed and analysed using a log-rank test. For all tests, a value of P < 0.05 was considered to indicate statistical significance. To discover the strength and direction of a link between two parameters, the Spearman’s rank correlation coefficient was calculated. A principal component analysis and dendrogram analysis was performed using the JMP software version 14 (SAS Institute). The random forest model was built using the random Forest package of the R software.

## Results

### *Bacteroides* contributes to faecal LPS levels

**Figure 2:**
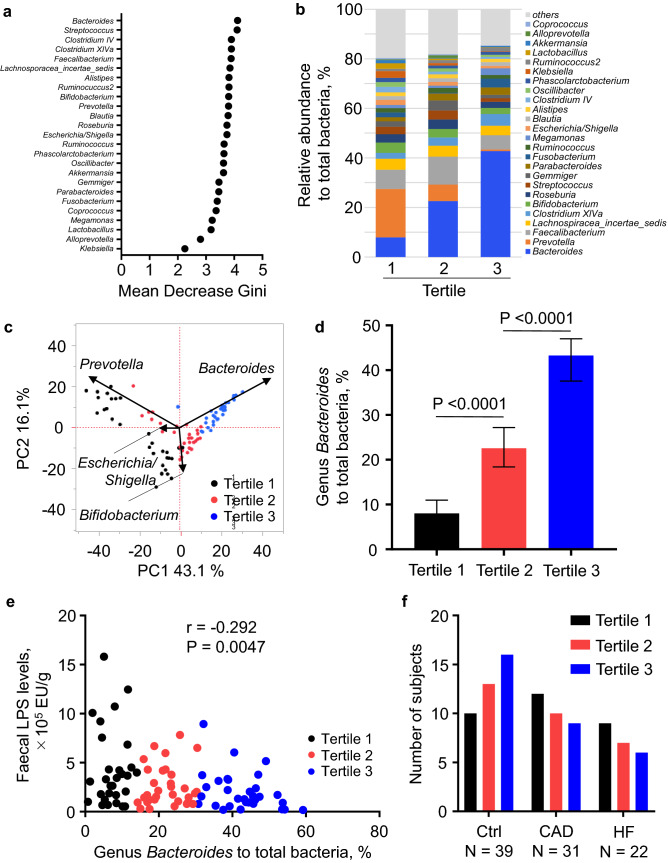
*Bacteroides* decrease faecal LPS levels. (**a**) Twenty-five genera of the gut microbiota were ranked according to the importance of their contribution to faecal LPS levels, as determined by a random forest classifier. (**b**) Patients were divided into three groups according to the abundance of *Bacteroides* in the gut microbiota. The relative abundance of the top 25 genera are indicated. (**c**) A principal coordinate analysis was performed to compare the distribution of each genus in the gut microbiota. Only genera with larger weight on principal coordinate analysis are shown. (**d**) Relative abundance of *Bacteroides* (percentage of total gut bacteria) among three groups. (**e**) Spearman’s rank correlation coefficient was calculated between faecal LPS levels and relative abundance of *Bacteroides*. (**f**) Distribution of controls and patients with CAD or HF in each cluster. Data are shown as median ± interquartile range (25th–75th percentile) (**d**). CAD, coronary artery disease; EU, endotoxin units; HF, heart failure; LPS, lipopolysaccharide.

To examine which gut microbiota contribute to faecal LPS levels, we used our previous two clinical data sets^1,30^. As shown in Fig. 1, the data of 92 patients (39 control patients, 31 CAD patients, and 22 HF patients) were analysed. We first used a random forest classifier to rank 25 gut microbiota organisms at the genus level, according to the importance of their contribution to faecal LPS levels. We found that the genus *Bacteroides* ranked first among the top 25 genera of the gut microbiota (Fig. 2a).

We then divided the study population into three groups according to the tertile of abundance of *Bacteroides* (Fig. 1). Baseline characteristics, including age, sex, body mass index, comorbidities, medications, and laboratory data are listed in Table 1. The age of patients in tertile 2 was significantly lower compared to the other groups (70.1 ± 2.1 (tertile 1) vs. 62.8 ± 2.1 (tertile 2) vs. 65.8 ± 1.7 (tertile 3), P = 0.04.). The plasma LPS levels were not different among the groups. No significant differences were observed in other characteristics among the three groups. The relative abundance of the top 25 genera detected in the gut microbiota in the three groups is shown in Fig. 2b. The principal coordinate analysis, at the genus level, showed that the three groups differed in the abundance of major gut bacteria, where tertile 1 showed a relative enrichment in the *Prevotella* and *Escherichia/Shigella* genera (Fig. 2c). The abundance of *Bacteroides* among the groups was significantly different (8.01% [range, 0.71–14.9%] vs. 22.55% [range, 15.90–30.48%] vs. 43.25% [range, 30.77–59.22%]; P < 0.0001.) (Fig. 2d). Of note, the faecal LPS levels in tertile 1 were significantly higher than those in tertile 3 (3.075 × 10^5^ EU/g [range, 0.313–15.810 × 10^5^ EU/g] vs. 1.523 × 10^5^ EU/g [range, 0.183–8.944 × 10^5^ EU/g]; P < 0.05.). Surprisingly, plotting faecal LPS levels with *Bacteroides* abundance revealed a significant negative correlation between these two parameters (r = − 0.292, P = 0.0047) (Fig. 2e). Although not significant, the control patients were more likely to be categorised into tertile 3, whereas the patients with CAD and HF were more likely to be categorised into tertile 1 or tertile 2 (Fig. 2f).

**Table 1:** Patients characteristics in Study A.

Variables	Tertile 1 (*Bacteroides* < 15.0%) N = 31	Tertile 2 (15.0% ≦ *Bacteroides* < 30.5%) N = 30	Tertile 3 (30.5% ≦ *Bacteroides*) N = 31	P value
Age, years	70.1 ± 2.1	62.8 ± 2.1	65.8 ± 1.7	0.04
Sex, male	22 (71)	24 (80)	24 (77)	0.69
Body Mass Index, kg/m^2^	25.1 ± 1.1	25.9 ± 0.8	24.3 ± 0.6	0.34
**Blood pressure, mmHg**				
Systolic	121.9 ± 2.9	119.2 ± 2.8	123.5 ± 2.8	0.58
Diastolic	67.1 ± 2.0	65.0 ± 1.7	67.1 ± 1.8	0.62
Smoking	19 (61)	17 (57)	22 (71)	0.50
**Comorbidities**				
Diabetes mellitus	16 (52)	8 (27)	11 (35)	0.13
Hypertension	29 (94)	25 (83)	25 (81)	0.31
Dyslipidemia	22 (71)	19 (63)	20 (65)	0.79
Atrial fibrillation	13 (42)	14 (47)	16 (52)	0.75
Coronary artery disease	12 (39)	10 (33)	9 (29)	0.72
Heart failure	9 (29)	7 (23)	6 (19)	0.67
**Medication**				
β-blocker	13 (42)	14 (47)	15 (48)	0.87
ACE-I/ARB	13 (42)	13 (43)	21 (68)	0.07
Calcium channel blocker	14 (45)	15 (50)	15 (48)	0.93
Antiplatelet	15 (48)	11 (37)	10 (32)	0.41
Anticoagulant	14 (45)	13 (43)	16 (52)	0.79
PPI/H2 blocker	25 (81)	22 (73)	19 (61)	0.23
Statin	16 (52)	16 (53)	16 (52)	0.99
**Laboratory data**				
Aspartate transaminase, U/L	25.1 ± 1.7	25.2 ± 1.4	25.5 ± 2.2	0.99
Alanine transaminase, U/L	23.1 ± 2.3	16.3 ± 3.2	21.3 ± 1.5	0.34
Blood urea nitrogen, mg/dL	20.5 ± 2.1	17.1 ± 1.0	19.6 ± 2.1	0.41
Creatinine, mg/dL	1.1 ± 0.06	0.9 ± 0.04	1.0 ± 0.07	0.30
Glycohemoglobin, %	6.5 ± 0.3	6.2 ± 0.2	6.3 ± 0.2	0.61
C-reactive protein, mg/dL	0.27 ± 0.1	0.66 ± 0.3	0.14 ± 0.04	0.16
Total cholesterol, mg/dL	171.8 ± 6.5	172.5 ± 5.5	184.7 ± 6.5	0.26
HDL-C, mg/dL	69.0 ± 6.8	65.9 ± 4.8	73.7 ± 6.6	0.67
LDL-C, mg/dL	81.4 ± 6.2	90.0 ± 6.9	90.7 ± 6.9	0.55
Triglycerides, mg/dL	133.2 ± 13.3	134.3 ± 13.8	140.8 ± 11.3	0.90
Plasma LPS, EU/mL	3.6 ± 0.3	4.0 ± 0.35	4.0 ± 0.4	0.65

Data are shown as mean ± standard error of the mean or n (%)

*ACE-I* angiotensin-converting enzyme inhibitor; *ARB* angiotensin receptor blocker; *HDL-C* high-density lipoprotein cholesterol; *LDL-C* low-density lipoprotein cholesterol; *PPI* proton pump inhibitor.

### *Bacteroides* LPS showed drastically low endotoxicity

We next extracted the *Bacteroides* LPS to examine its biological activity and compare with *E. coli* LPS, which has a strong biological activity. We cultured *B. dorei* and *B. vulgatus*, the two most abundant *Bacteroides* species in human gut microbiota^1^^,^ and purified LPS from these species. The biological activity of LPS was evaluated by three different methods: endotoxin units via LAL tests, cytokine secretion from RAW cells, and survival rate after intraperitoneal injection of mice.

**Figure 3:**
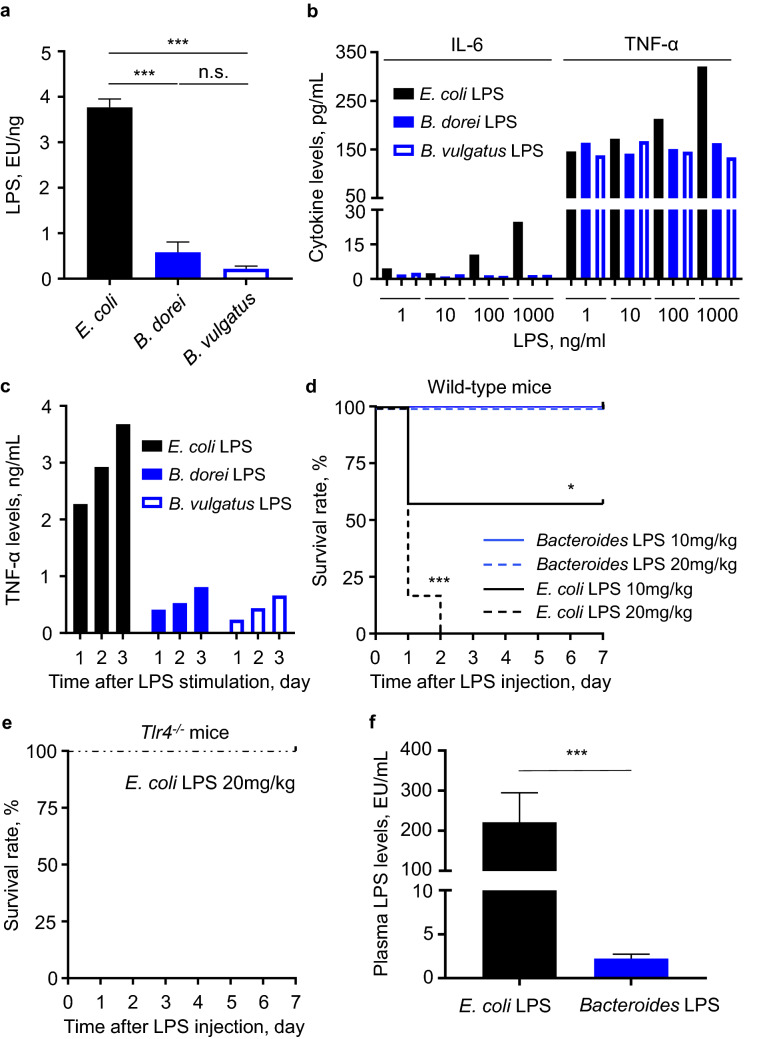
*Bacteroides* LPS shows lower LAL activity and immunogenicity. (**a**) LAL activity of the indicated LPS preparation (1 ng) was measured. The results were the sum of three independent experiments. (**b**) RAW 264.7 cells were stimulated with the indicated LPS preparation for 12 h. The cytokine levels in supernatants were quantified. (**c**) RAW 264.7 cells were stimulated with the indicated LPS (10 ng/mL) for 24, 48, and 72 h. The cytokine levels in supernatants were quantified. (**d**) Eight-week-old wild-type mice were treated with LPS intraperitoneally to induce septic shock. *Bacteroides* LPS consisted of half each of *B. dorei* LPS and *B. vulgatus* LPS. Survival was monitored daily. The log-rank test was used to determine statistically significance. N = 5 to 8 per group. (**e**) Eight-week-old toll-like receptor 4-deficient (*Tlr4*^−/−^) mice were treated with *E. coli* LPS intraperitoneally. N = 5. (**f**) Plasma LPS levels 12 h after LPS injection intraperitoneally. *Bacteroides* LPS consisted of half each of *B. dorei* LPS and *B. vulgatus* LPS. N = 5 per group. EU; endotoxin units, LAL; Limulus amoebocyte lysate. LPS, lipopolysaccharide. *P < 0.05, ***P < 0.001.

The results of the silver staining and Coomassie brilliant blue staining indicated that the purified *Bacteroides* LPS was structurally different from the *E. coli* LPS (Fig. S1). The *Bacteroides* LPS endotoxin units, as measured by the LAL tests, were remarkably lower than those measured for the *E. coli* LPS (Fig. 3a). RAW cells stimulated with the *Bacteroides* LPS produced lower cytokines when stimulated with higher LPS concentration. Moreover, stimulation of RAW cells with the *E. coli* LPS induced a dose-dependent increase in the secretion of pro-inflammatory cytokines IL-6 and TNF-α, whereas the secretion of cytokines did not increase with the *Bacteroides* LPS concentration (Fig. 3b). Interestingly, both *E. coli* LPS and *Bacteroides* LPS showed inflammatory potency in a time-dependent manner (Fig. 3c). Furthermore, dose-dependent sepsis-related death was observed in mice injected with *E. coli* LPS (10 mg/kg group, 43%; 20 mg/kg, 100%) by day 7 after injection (Fig. 3d). In contrast, none of the mice injected with the *Bacteroides* LPS or the *Tlr4*^−/−^ mice injected with *E. coli* LPS died (Fig. 3d,e). Plasma LPS level in mice injected with *E. coli* LPS, as measured by the LAL tests, was significantly higher than that in mice injected with *Bacteroides* LPS (221.2 ± 73.5 EU/mL vs. 2.2 ± 0.5 EU/mL, P < 0.001) (Fig. 3f)..

### *Bacteroides* probiotics increased the abundance of *Bacteroides* in a gut model

**Figure 4:**
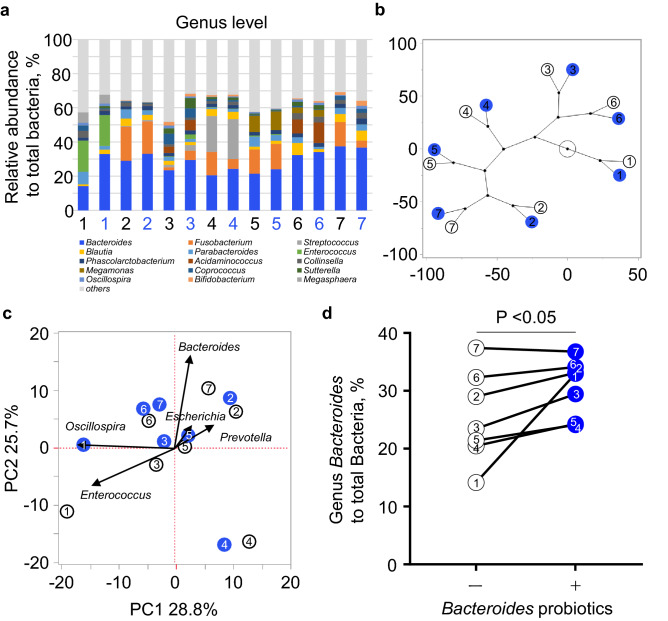
Alteration of the gut microbiota in the KUHIMM. The V3–V4 regions of the bacterial 16S rRNA gene in faecal sample cultures from seven patients were sequenced.(**a**) Relative abundance of the top 15 gut bacterial genera in faecal sample cultures from seven patients without (black letters) or with (blue letters) *Bacteroides* probiotics. (**b**) Dendrogram analysis at the genus level without (white circle) or with (blue dots) *Bacteroides* probiotics. (**c**) Principal component analysis score plots at the genus level without (white circle) or with (blue dots) *Bacteroides* probiotics. (**d**) The abundance of the genus *Bacteroides* without (white circle) or with (blue dots) *Bacteroides* probiotics. The Wilcoxon signed-rank test was used to compare the matched-pair samples. KUHIMM, Kobe University Human Intestinal Microbiota Model.

Finally, we simulated gut microbiota alteration in the KUHIMM to investigate whether *Bacteroides* probiotic (*B. dorei* and *B. vulgatus*) administration could increase the *Bacteroides* abundance in the human gut. We fermented the patient faecal samples with or without *Bacteroides* probiotics in the KUHIMM and then evaluated the microbiota. The mean age and body mass index of seven patients were 72.7 ± 2.2 and 24.2 ± 0.6 (Table 2). All patients had CAD with coronary risk factors. The relative abundance of the top 15 genera detected in the gut microbiota of the two groups is shown in Fig. 4a, which reveals the gut microbial alteration induced by *Bacteroides* supplementation. To visualise the impact of *Bacteroides* probiotics, we performed dendrogram analysis and found that *Bacteroides* probiotics changed the global composition of the gut microbiota to a certain extent, but the effect did not induce a drastic change in the composition of any single flora (Fig. 4b).The principal coordinate analysis at the genus level suggested increased *Bacteroides* abundance after *Bacteroides* probiotics supplementation (Fig. 4c). The abundance of *Bacteroides* was significantly higher with *Bacteroides* probiotics supplementation (30.7% [range, 24.1–36.8%]) than with non-*Bacteroides* probiotics supplementation (25.5% [range, 14.2–37.4%], P < 0.05.), though one faecal sample (No. 7) showed decreased abundance of *Bacteroides* after probiotics supplementation (Fig. 4d).

**Table 2:** Patients characteristics in Study C.

Variables	N = 7
Age, years	72.7 ± 2.2
Sex, male	7 (100)
Body Mass Index, kg/m^2^	24.2 ± 0.6
**Blood pressure, mmHg**	
Systolic	130.6 ± 3.0
Diastolic	65.4 ± 3.0
**Comorbidities**	
Diabetes mellitus	6 (86)
Hypertension	7 (100)
Dyslipidemia	6 (86)
**Medication**	
β-blocker	4 (57)
ACE-I/ARB	6 (86)
Calcium channel blocker	4 (57)
Antiplatelet	7 (100)
PPI/H2 blocker	7 (100)
Statin	5 (71)
**Laboratory data**	
Aspartate transaminase, U/L	27.0 ± 2.4
Alanine transaminase, U/L	24.7 ± 5.5
Blood urea nitrogen, mg/dL	18.2 ± 1.5
Creatinine, mg/dL	0.92 ± 0.04
Glycohemoglobin, %	6.5 ± 0.2
C-reactive protein, mg/dL	0.21 ± 0.05
Total cholesterol, mg/dL	161.0 ± 18.9
HDL-C, mg/dL	46.7 ± 5.4
LDL-C, mg/dL	91.4 ± 14.1
Triglycerides, mg/dL	144.7 ± 30.4

Data are shown as mean ± standard error of the mean or n (%).

*ACE-I* angiotensin-converting enzyme inhibitor; *ARB* angiotensin receptor blocker; *HDL-C* high-density lipoprotein cholesterol; *LDL-C* low-density lipoprotein cholesterol; *PPI* proton pump inhibitor.

## Discussion

Many clinical and basic scientific reports have stated that faecal LPS, which is derived from the gut microbiota, has a clinical impact on patients with irritable bowel syndrome, autoimmune disease, and cardiovascular disease^1,13,19,22^. However, experimental studies have yet to delineate the impact of specific gut bacteria on faecal LPS levels. Furthermore, though LPS-lipid A structure differs between bacteria, the impact of LPS-lipid A structural differences on faecal LPS levels has not yet been measured using the LAL tests. In the present study, we indicated that the abundance of the genus *Bacteroides* is negatively correlated with the faecal LPS levels measured by the LAL tests. Taken that the LPS purified from the two species of *Bacteroides* showed low biological activity in the LAL tests, it is logical that the increased abundance of *Bacteroides* should exhibit lower faecal LPS levels.

*Bacteroides* is one of the predominant genera in the human gut and plays an important role in maintaining the stability of a healthy gut ecosystem^39^. Interestingly, the LPS component, lipid A, which activates TLR4, exhibits structural variation. The *Bacteroides* species do not express *lpxM*, which yields the hexa-acylated lipid A moiety found in *E. coli*; instead, the lipid A component of *Bacteroides* LPS are penta- or tetra-acylated^13,14^. The penta- and tetra-acylated lipid A moieties are known to elicit reduced TLR4 responses^13,14^, this is consistent with our findings and suggests that the penta- or tetra-acylated *Bacteroides* LPS provides a beneficial effect on the progression of inflammatory diseases. We also investigated the impact of LPS structural variation on the LAL activity and found that the *Bacteroides* LPS demonstrated significantly lower LAL activity compared to the *E. coli* LPS. Of note, the mice injected with *Bacteroides* LPS did not develop sepsis, indicating that the *Bacteroides* LPS did not exhibit endotoxicity in vivo. These results further suggest that the decreased abundance of *Bacteroides* in the gut microbiota might predispose people to more inflammatory diseases. Importantly, our results imply that it would be insufficient to analyse the gut metagenome data to evaluate LPS biosynthesis in the gut; LPS immunogenicity should be considered while interpreting the gut microbial LPS data.

We also showed that the administration of *Bacteroides* as probiotics in the KUHIMM manipulated the human gut microbiota toward increasing the abundance of *Bacteroides* in all except one sample (Fig. 4d). This results indicate that *Bacteroides* prebiotics did not always increase the abundance of *Bacteroides* as the human gut is home to trillions of microorganisms. Each gut microbial profile depends on age, sex, local food or lifestyle, drugs, and other many factors^8^. As the KUHIMM is designed to monitor changes in the bacterial composition and facilitates evaluations of the impact of probiotics in independent vessels, culture samples in the KUHIMM are unaffected by such factors. In that sense, we believe that the KUHIMM might be useful for carrying out preclinical evaluations of the effect of *Bacteroides* prebiotics on the gut microbiota. Our results at least suggest that the gut microbial alterations aimed at preventing and treating inflammatory diseases could be feasible in humans. These data could therefore lead to a novel strategy for the prevention and treatment of inflammatory diseases via manipulation of the gut microbiota.

From a clinical perspective, our results pave the way for further studies investigating faecal LPS levels and *Bacteroides* abundance. It was interesting that patients with CAD or HF were more likely categorised in tertile 1, which showed less *Bacteroides* abundance. A previous cross-sectional study also shows that the abundance of *Bacteroides* is lower in patients with atherosclerotic cardiovascular disease compared to healthy controls^9^. A cohort study is warranted to assess causality and to provide the strongest scientific evidence of the relationship between *Bacteroides* abundance and faecal LPS levels and disease progression; this will aid deeper understanding and acceptance of the importance of *Bacteroides* abundance and faecal LPS levels in human diseases. Further research is necessary to understand whether gut microbial composition directly reflects faecal LPS composition. Advances in mass spectrometry technology might expand our knowledge of LPS composition.

Fig. S1 shows the schematic illustration of the present study. In summary, we identified the *Bacteroides* genus as the primary constituent of the human gut microbiota and determined that the abundance of organisms of this genus was negatively correlated to LPS endotoxicity. We speculate that this reduced immunogenicity is linked to the lower levels of cytokine stimulation and, therefore, *Bacteroides* might present a valuable therapeutic option in certain inflammatory conditions. We also demonstrated that supplementation with *Bacteroides* as a probiotic in a model gut system increased the abundance of this genus.

## Supplementary information

Supplementary figures.

## Data Availability

All data supporting the findings of our study are available from the corresponding author upon reasonable request. The sequencing data were deposited to the Japan BioProject database with links to the BioProject accession number PRJDB8454.
