# Supplementary material for: A possible beneficial effect of Bacteroides on faecal lipopolysaccharide activity and cardiovascular diseases
Source: Sci Rep. 2020 Aug 3;10:13009. doi: 10.1038/s41598-020-69983-z (PMC7398928; doi:10.1038/s41598-020-69983-z)
Supplement: Supplementary file 1 — Supplementary figures. [file 41598_2020_69983_MOESM1_ESM.pdf]

Supplementary Information: A possible beneficial effect of *Bacteroides* on faecal lipopolysaccharide activity and cardiovascular diseases

Naofumi Yoshida[a], Tomoya Yamashita[a], Shigenobu Kishino[b], Hikaru Watanabe[c], Kengo Sasaki[d], Daisuke Sasaki[d], Tokiko Tabata[a], Yuta Sugiyama[b], Nahoko Kitamura[b], Yoshihiro Saito[a], Takuo Emoto[a], Tomohiro Hayashi[a], Tomoya Takahashi[c], Masakazu Shinohara[e], Ro Osawa[f], Akihiko Kondo[d], Takuji Yamada[c], Jun Ogawa[b], Ken-ichi Hirata[a]

[a]Division of Cardiovascular Medicine, Department of Internal Medicine, Kobe University Graduate School of Medicine, Kobe 6500017, Japan

[b]Division of Applied Life Science, Graduate School of Agriculture, Kyoto University, Kyoto 6068502, Japan

[c]School and Graduate School of Bioscience and Biotechnology, Tokyo Institute of Technology, Tokyo 1528550, Japan

[d]Graduate School of Science, Technology and Innovation, Kobe University, Kobe 6578501, Japan

[e]Division of Epidemiology, Department of Community Medicine and Social Healthcare Science, Kobe University Graduate School of Medicine, Kobe 6500017, Japan.

[f]Department of Bioresource Science, Graduate School of Agricultural Science, Kobe University, Kobe 6578501, Japan

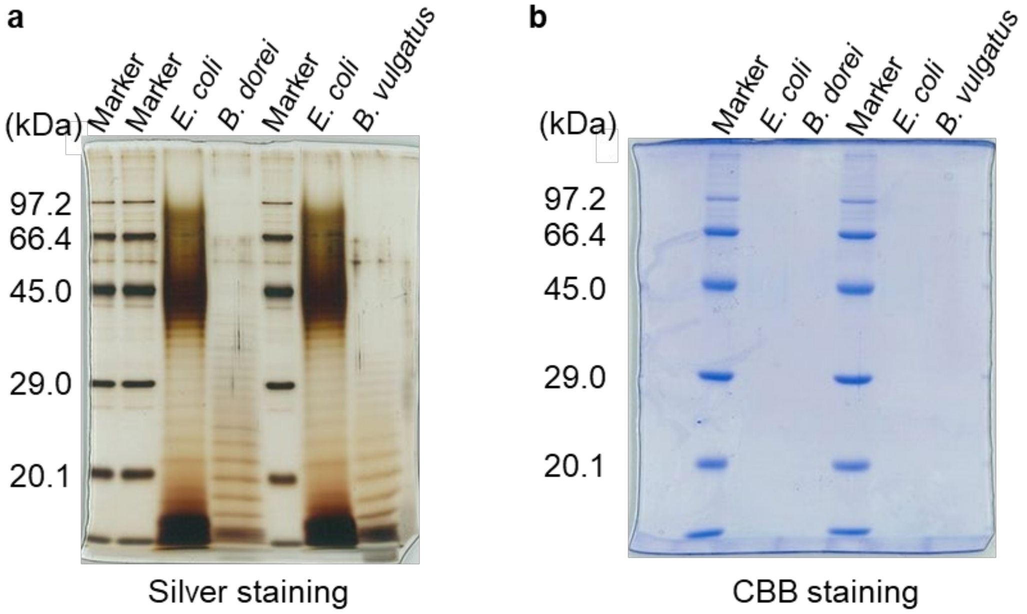

Supplementary Figure 1. Profiles of silver-stained samples of *Bacteroides* and *Escherichia coli* LPS on polyacrylamide gels

(A) LPS samples of 15 µg per well were subjected to SDS-PAGE electrophoresis. The silver staining was performed to detect LPS. (B) The results of the Coomassie Brilliant Blue staining shows that the LPS extracts were well purified. CBB, Coomassie Brilliant Blue; LPS: lipopolysaccharide; SDS-PAGE: sodium dodecyl sulphate-polyacrylamide gel electrophoresis.

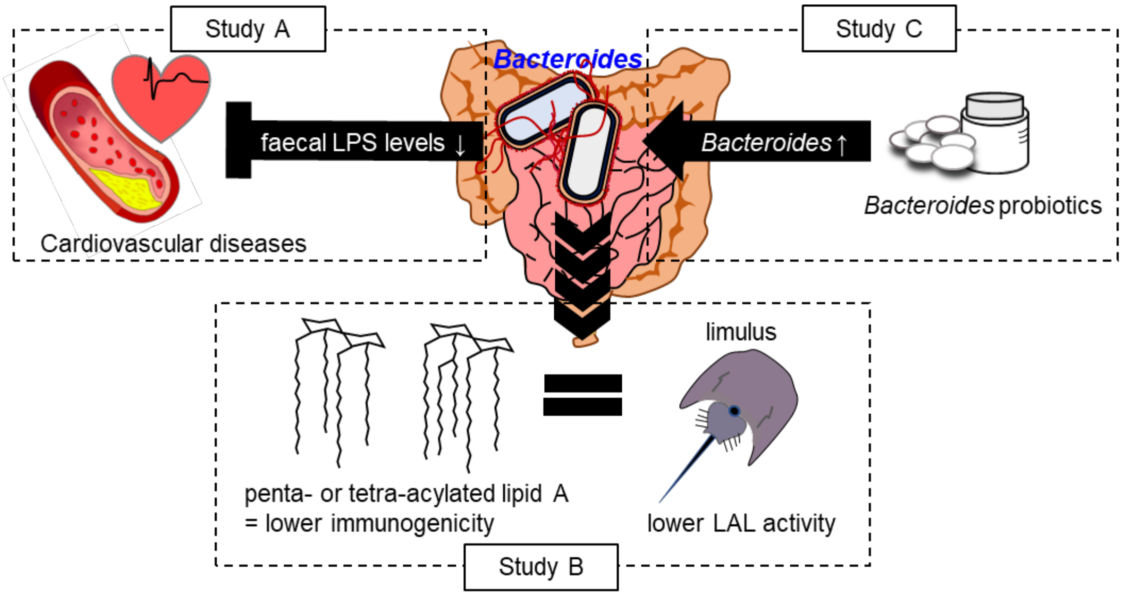

Supplementary Figure 2. Schematic illustration of the present study

Study A, B, and C correspond to Figure 1.

LAL: limulus amoebocyte lysate, LPS: lipopolysaccharide.
